# Sexsomnia: case based classiﬁcation and discussion of psychosocial implications

**DOI:** 10.5935/1984-0063.20200057

**Published:** 2021

**Authors:** Andrea Cecilia Toscanini, Julia Hatagami Marques, Rosa Hasan, Carlos H. Schenck

**Affiliations:** 1 Faculdade de Medicina Universidade de Sao Paulo, LIM-63 - Sao Paulo - SP - Brazil.; 2 Hospital das Clínicas da Universidade de Sao Paulo, Instituto de Psiquiatria - Sao Paulo - SP - Brazil.; 3 Hennepin County Medical Center and University of Minnesota Medical School, Minnesota Regional Sleep Disorders Center and Department of Psychiatry - Minnesota - Minneapolis - United States.

**Keywords:** Parasomnias, Sexual Behavior, Family Relations, Sexsomnia, Sleep Sex, NREM Parasomnia

## Abstract

We describe a 42-year-old married woman diagnosed with sexsomnia as a NREM parasomnia, who sought medical assistance motivated by relationship problems with her husband after two sexsomnia episodes. This is the second case of sexsomnia reported in Brazil, but the first case with comprehensive follow-up. The patient was clinically evaluated, no psychiatric history was found, and she denied using pharmaceutical or recreational drugs. A video-polysomnography documented nine episodes of short- lasting abrupt awakening from N2 and N3, indicating a non-REM parasomnia, some with masturbation characteristics. The findings of this case, including unusual features, are considered in regard to the range of adverse psychosocial consequences of sexsomnia in these patients and the need for specialized interventions that can be provided by sleep specialists. We discuss the misinformation and delay of proper diagnosis and treatment that occurs with sexsomnia and emphasize the importance of understanding the broad set of problems and consequences related to sexsomnia, including physical, psychological, marital/relationship and at times legal aspects that affect the lives of sexsomniac patients and their bed partners.

## INTRODUCTION

Parasomnias are classified as undesirable physical events, experiences, and autonomic nervous system activity that occur during any stage of sleep or its transitions from or into wakefulness. They are mainly divided in those which occur in non-rapid eye movement (NREM) and rapid eye movement (REM) sleep (ICSD3)^1^. Sexsomnia, characterized by sexual behavior during sleep, is within the spectrum of parasomnias occurring predominantly in NREM sleep, as a variant of confusional arousals and sleepwalking, with or without associated obstructive sleep apnea^1^. It can vary from sleep masturbation to sexual moaning and vocalizations, to fondling and full sexual intercourse with a bed partner. In all reported cases, memory of the sexual event is completely or almost completely impaired^2^^-^^6^.

Little information exists regarding the epidemiology of sexsomnia in the general population, probably because most individuals are unaware of sexsomnia as a medical issue and only look for medical assistance when facing negative consequences^7^. Also, embarrassment may prevent many people from seeking help^8^.

The unconscious sleep-related sexual behavior often leads to adverse psychosocial impacts for the subjects and bed partners, and sometimes can lead to physical injuries and sexual assaults^2^^,^^3^^,^^5^^,^^6^. Although the forensic consequences of sexsomnia have been reported^9^, the psychosocial impact of sexsomnia on patients and bed partners needs to be more fully understood.

Here we report a case of a 42-year-old woman, with a previous history of sleep talking during childhood, who developed sexsomnia during her marriage. In recent years, it became a problem for her marital relationship, for the husband’s self-esteem, and for her young son who witnessed an episode. Based on this case, and a focused literature review, we aim to discuss sexsomnia and propose a systematic classification for its psychosocial implications.

## CASE REPORT

In July 2015, we received in our outpatient sleep medicine practice a 42-year-old woman, whose husband complained about her abnormal sexual behavior during sleep. She had no memory of the episodes and no dreams related to them. This was first noticed in 2005 by her husband, and has occurred, since then, in an occasional pattern of up to twice monthly. It took five years for her to believe that she actually engaged in such nocturnal behaviors, so it was only in 2010 that she sought medical assistance. She consulted with a primary care doctor and a neurologist, who could not identify the basis for those symptoms, and after epilepsy was excluded, she was referred to our hospital psychiatry practice. The patient reported sleep talking since she was 12-years-old, but she had no memory about these events, and she claimed to have no other sleep problems during childhood. In 1999 she remembers two confusional awakenings when she woke up with her own voice. No other unwanted event during the night was reported by the patient during the anamnesis. According to the patient’s report, she developed sexsomnia during her marriage. She presented no psychiatric history, nor any mental disorder at the time of evaluation, and then she was referred to the sleep medicine group.

According to her husband, she usually moaned in a sexual manner in her sleep and uttered the names of men and objects (not related to sexual behaviors, such as “table”), phrases involving sexual content and “dirty talk” which she never said while awake. In some episodes she also masturbated. There had been episodes in which she fondled her husband, who then engaged in sexual activity with her. In the middle of it, she woke up feeling somewhat abused, because from her point of view he was forcing sexual intercourse during her sleep without consent. This was very unpleasant for her and led to many arguments, mistrust and distancing between the couple. The husband was feeling insecure over not satisfying her sexually and sometimes he thought she could be betraying him with other men, since she uttered the names of other men in her sleep while acting in a sexual manner.

The search for professional help was motivated by the mistrust of her husband, after she masturbated in sleep while saying the name of a coworker. Another motivator was an episode witnessed by her 9-year-old son, who heard her moaning sexually out loud.

She had a previous history of two sleep talking episodes without sleepwalking witnessed by her siblings. After becoming married at the age of 24, her husband witnessed two episodes of confusional arousals during which she “woke up” while being verbally aggressive and shouting nonsensical phrases at him.

At the time of first consultation, she denied the use of pharmaceutical or recreational drugs besides contraceptive pills, and there was no history of alcohol abuse. She worked with information technology and reported considerable stress at work, related to deadlines, and pressure from her boss to complete many tasks on her own in a short period of time. She went to bed around 9 p.m. and woke up at 6 a.m., feeling tired. She snored 3-4 times per week, with no apnea episodes reported by her husband.

Her physical examination found obesity, with a body mass index (BMI) of 31, and a modified Mallampati Class IV (this test is based on visual examination of oral cavity)^10^. Class IV indicates that the space between tongue base and roof of mouth was very small and only the hard palate is visible^11^^,^^12^, suggesting a higher chance of obstructive sleep apnea (OSA). The rest of her physical exam was within normal parameters.

The results of the Berlin questionnaire, a screening test for OSA based on snoring, tiredness/sleepiness, blood pressure and BMI, suggested a high risk for OSA. The Epworth sleepiness scale score was 13/24, indicating excessive daytime somnolence^13^^,^^14^.

A waking electroencephalogram (EEG) showed instability of cerebral electrical activity in temporal regions, bilaterally, with predominance on the left, mainly during wakefulness, with no clinical significance. Brain magnetic resonance imaging and routine blood tests were unremarkable.

We performed a one-night time-synchronized video-polysomnographic (VPSG) study that continuously monitored her EEG (F3-M2, C3-M2, O1-M2, F4-M1, C4-M1, O1-M2), electrooculogram, electromyography of submentalis and anterior tibial regions, position in bed, snoring, oral and nasal airflow, abdominal and thoracic respiratory effort, peripheral hemoglobin oxygen saturation and one- derivation electrocardiogram. Results from VPSG are summarized below:

- There were no relevant respiratory events during the night (AHI = 3.3/hour);
- Total recording time: 397 minutes;
- Total sleep time: 367 minutes;
- Sleep onset latency: 10.4 minutes;
- REM onset latency: 85 minutes;
- Sleep efficiency: 92.5%;
- N1 = 8%, N2 = 46%, N3 = 23.2%, REM = 22.8%;
- Wake after sleep onset (WASO): 19.2 minutes;
- Arousal index: 7.8/hour.

**Figure 1:**
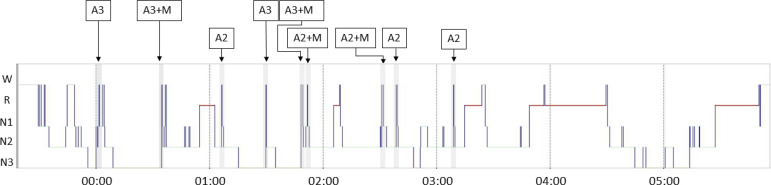
vPSG hipnogram showing abrupt arousals. A3: awakening from N3. A2: awakening from N2. +M: association with motor behavior of limbs and hips that resembled masturbation together with a mixture of moaning and sighing.

The VPSG documented nine episodes of short-lasting abrupt awakening from N3 (4) and N2 (5), some of them with quasi-stereotyped motor behavior of the limbs and hips and moaning, compatible with confusional arousals (Figure 1). Characteristics that resembled masturbation (contraction of thighs and arms moving towards her pelvis) together with a mixture of moaning and sighing were observed in two awakening from N2 and two from N3. These findings indicated a non-REM parasomnia.

A video-EEG was then performed for 4 days continuously during sleep and wakefulness. The international 10-20 system EEG montage was used, with electrocardiogram electrodes in addition. VEEG showed adequate basal brain electric activity, together with instability of cerebral electric activity in temporal regions, predominantly on the left side and during wakefulness, with no clinical significance. During approximately 6 hours of sleep each night, she presented 4 to 5 episodes suggestive of confusional arousals with nonsensical movements and discrete masticatory non- stereotyped movements, but no sexual events were observed during that recording period. EEG showed no epileptiform correlate during the arousal episodes.

Treatment with clonazepam up to 0.6mg/bedtime improved her daytime somnolence but had no effect on the sexsomnia. Around one year after the first consultation, her life situation changed drastically. She had suspended the use of clonazepam on her own because it had no effect on sexsomnia. She was transferred to another area at her work in which she had almost no stress. Health problems in a family member requiring care reduced the duration of her nocturnal sleep to 4.5 hours daily, with no reported excessive daytime somnolence.

Curiously, the sexsomnia episodes and the daytime somnolence ceased from this period onward. Currently, she sleeps for 6 to 7 hours per night, with no daytime somnolence nor episodes of sexsomnia, according to her husband, up to the latest follow-up in December 2018. The patient reports that cessation of her sexsomnia episodes and the explanation to the couple that sexsomnia is a sleep disorder, helped reunite the couple and reestablish their prior level of intimacy.

## DISCUSSION

This is the second Brazilian case of sexsomnia published. In the first case, a 27-year-old man, during sexsomnia episodes, typically searched for his wife, “achieving complete sexual intercourse with total amnesia… His wife remained in bed with him after the episodes”. Therefore, the previous Brazilian case involved a male who engaged in sexual intercourse with his wife during sleep, in contrast to our case that involved a woman with sleep masturbation and vocalizations^15^.

This case report describes a 42-year-old married woman with a prior history of sleep talking but without a history of sleepwalking, who was diagnosed with sexsomnia as a NREM parasomnia with confirmatory vPSG. There was no comorbid OSA.

The search for medical help was motivated by relationship problems between the patient and her husband after two episodes; the first occurred when she masturbated during sleep while saying the name of another man (a coworker), and the second episode occurred when her 9-year-old son heard her moaning sexually out loud. These compelling situations within the familial nucleus of these patients, deserves special attention regarding the distinctly adverse psychosocial impact of sexsomnia.

As previously described by Dubessy et al.^6^, in a series of 17 cases, especially in female patients, marital difficulties are common, including feelings of strangeness, guilt, shame or depression (one of the patients left a suicide letter and went to jump in a river, but renounced)^6^. Additionally, one sexsomnia patient in this French series, a 76-year-old married woman, engaged in repeated episodes of sleep masturbation while uttering sexual vocalizations - and calling out the name of a man, Mike, who was not her husband (similar to our case), and she denied knowing any man named Mike^6^. This caused great marital stress, and this nocturnal scenario was identical to that of our reported patient. Sleep talking is non-volitional and does not reflect waking mentation, and so it is not admissible evidence in any legal proceedings^9^, nor should it be regarded as volitional behavior or “subconscious” motivation in a clinical setting. Considering these sexsomnia-related negative scenarios, a systematic therapeutic approach should be used for patients and spouses, and other relatives.

### Misinformation and delay of proper diagnosis and treatment

Sexsomnia was first described in a case from Singapore, in 1986, involving nightly sleep masturbation in a married man, which made his wife feel inadequate, especially since they had nightly intercourse before falling asleep^16^. In ICSD3^1^, released in 2014, it is classified as a subtype of confusional arousals, named sleep related abnormal sexual behavior. In Brazil, this is the second female case of sexsomnia reported in the literature to date.

The majority of previously reported sexsomnia cases involve middle-aged men, with a previous history of other NREM parasomnias. In most cases, the events occurred in bed or within the sleep accommodation, but some reports documented events in other rooms, what may be related to sleepwalking. It is not known yet if this male predominance is due to a gender-determined predisposition or due to bias in seek for medical assistance.

Various factors influence the search for professional help. First of all, a bed partner is needed for sexsomnia to be noticed, otherwise the person affected by sexsomnia may never know (about this condition), because of the inherent amnesia. Second, a problem is identified in cases where either there is a bed partner who suffers in some way or the “sexsomniac” repeatedly hurts oneself physically from vigorous sleep masturbation. Moreover, there is often considerable embarrassment involving sexsomnia, which prevents people from talking to a doctor about it^8^^,^^17^. Also, some people may not believe it is a medical problem, and for that reason do not seek medical attention.

Most people, including health professionals, are not aware that this is a sleep disorder. Therefore, few patients seek professional help and, when they do, some of them end up with no clinical diagnosis, and are referred to psychotherapy due top resumed psychological issues promoting the sexsomnia. Conversely, some patients and partners may need psychotherapy to deal with the consequences of sexsomnia. This can result in substantial delays in proper diagnosis and correct treatment, perpetuating suffering for the sexsomniac and for bed partners or people who live in the same house.

### Addressing problems and consequences as part of treatment

It is well established that sexsomnia may cause many problems from physical to legal, as well as psychosocial problems for the affected person and for bed partners or people living/sleeping in the same house/place. Pleasant feelings for bed partners, although rare, have also been described^2^. We list and categorize all ascribed problems/benefits of sexsomnia and their consequences (Table 1).

**Table 1:** Problems/ benefits.

Event	Sexsomniac		Bed partner		People in the house/dormitory	
Sleep superficialization (meaning?) /Arousals	Disrupted sleep	(F)				
Self masturbation	Briuses/physical injuries	(F)	Disrupted sleep	(F)		
			Psychological insecurity (regarding not satisfying partner sexually and concern over extramarital affairs)	(P)		
			Bruises/physical injury	(F)		
Masturbation of other	Sexual abuse prosecution	(L,R)			Sexual abuse/child abuse	(F,P,R)
sleepsextalking or vocalizations	Accusations of infidelity	(PR)	Disrupted sleep	(F)	Disrupted sleep or worry	(F,P)
			Hearing offensive phrases, psychological discomfort: feelings of shock, worry, alarm, anger, annoyance, bewilderment	(P)		
			Psychological insecurity, suspicion of extramarital affairs (names of others uttered)	(P,R)		
Intercourse attempt			Disrupted sleep	(F)		
			Inappropriate time for sex	(P)		
			Inappropriate type of sex	(P,F)		
Sexual intercourse	Physical injuries Sexual abuse/rape prosecution	(F)	Physical injuries	(F)		
	Awakening in the middle of intercourse and feeling abused	(L,R)	Rape-like experiences Inappropriate type of sex	(F,P,R)		
	Nonconsensual intercourse (asleep)	(F,P,R)	Psychological confusion, feelings of rejection and frustration. Accusation of sexual abuse	(P,R)		
			More pleasurable sex ("kinky sex or less hurried sex, even with bruises ometimes) (F,P)	(F,P)		
			More gentle, amorous lover, more oriented towards satisfying female partner when asleep			
			Negative relationship impact	(P,R)		
Communication of sexsomnia events (bed partner telling sexsomniac)	Denial	(P)	Negative relationship impact			
	Worries for not controlling own body, feelings of guilty, shame, inadequacy. Confusion and despair related to amnesia for the event: being told about one`s objectionable sleep behaviors after awakening in the morning	(P)		(P,R)		
	Opportunity for seeking professional help	(F,R,P)	Opportunity for seeking professional help	(F,R,P)		
Diagnostic	Emotional relief	(P)	Emotional relief, understanding	(P)		
	Possibility of treatment	(F,P)	Positive relationship impact	(P,R)		

Physical (F), psychological (P), relational (R), legal (L).2,9.

When treating sexsomnia it is important to approach all physical (F), psychological (P), relational (R) and sometimes legal (L) aspects that affect the lives of sexsomniac patients and bed partners. The presence of the partner at consultation is essential since she or he is the one consciously witnessing and experiencing sexsomnia. Health professionals should not feel embarrassed when talking about sexsomnia nor with patients and partners sexual life.

At first consultation, a simple psychoeducational intervention may resolve many of the patients and partners negative psychological consequences and relational problems. It is important for them to understand that sexsomnia or sleep sex talking events are considered to be a sleep disorder that occurs unconsciously and does not depend on desire for other people, sexual dissatisfaction, need for sex or infidelity. When a bed partner is experiencing pleasant feelings from sexsomnia, it is also important to explain the necessity for treatment and remission of symptoms, since these feelings are usually mixed with the negative consequences in the clinical setting, prompting the referral.

Investigating the emotional profile of each individual and the relationship of the patient and bed partner may sometimes be necessary, in order to address negative feelings such as guilt, insecurity, mistrust, and resentment due to accusations. Individual and couples/family therapy can be considered in some cases, when negative psychological consequences do not recede after adequate information or when the relationship between sexsomniac patient and other people has become compromised.

Medication therapy should be offered to prevent events and to regulate sleep architecture of patients. The pharmacological treatment with clonazepam had no effect on the parasomnia complaints. Clonazepam is commonly used as first line pharmacotherapy for sexsomnia^2^^,^^4^. Also, in 1996, there was a report on a series of 170 patients with various parasomnias treated with benzodiazepines, primarily clonazepam (n=136)**,** and followed long-term for clinical response, which showed that the vast majority of all patients (86%) reported good control after an average follow up of 3.5 years^18^. On the other hand, another report claimed that clonazepam failed to demonstrate sustained efficacy in 5 sleepwalking patients. This investigation carefully excluded even subtle sleep disordered breathing. After 1 year, all patients treated with clonazepam dropped out of the study and reported a persistence of sleepwalking^19^.

There is evidence concerning the use of melatonin in REM sleep behavior disorder as a second line treatment with an overall improvement, reduced injuries and less side effects, as compared with clonazepam^20^. But, a recent retrospective review on NREM parasomnias in 512 patients discussed therapeutic options for these conditions, including melatonin. Among these patients, there were 15 cases of sexsomnia**,** and in nine of these cases, they used benzodiazepines, in three cases they treated with CPAP, and in one case they treated with non-pharmacological approaches; however, melatonin was not used^21^. Therefore, in the absence of available evidence, we did not try any other pharmacological approach.

Other sleep disorders are frequently associated with, and contribute to NREM parasomnias. Obstructive sleep apnea (OSA) and restless legs syndrome (RLS) with periodic limb movements of sleep are the most commonly identified precipitating factors in patients with sleepwalking^22^. Other conditions that are associated with NREM parasomnias are also characterized by sleep fragmentation and/or increased homeostatic sleep pressure, including shift work, sedatives, environmental sleep disruption, PLMs, and gastro-esophageal reflux^23^^-^^25^.

Also, in cases of sexsomnia resulting from OSA, therapy with CPAP or a mandibular retaining device may be effective in reducing sexsomnia symptoms and the adverse physical and psychosocial consequences^26^^-^^28^. This will also prevent disruption of sleep of bed partners and perhaps other people in the house. Once the episodes cease, all physical consequences cease as well.

Even when medicated, patients must be oriented about measures to guarantee the security of everyone sleeping in the same room and house in order to prevent physical injuries and psychological consequences and also legal consequences^29^, especially when minors live or travel with a sexsomniac. It is advisable not to sleep in the same room with other people, mainly with children/minors. Although no episode of pregnancy was reported, it is important to remember that women with sexsomnia at fertile age are at risk for unwanted pregnancy during episodes involving complete intercourse with male partners. They should be advised to search for the most adequate contraceptive method together with her gynecologist.

We believe this case report presents individual perspectives regarding the relationship between two people in both a personal and physical context as well as the human ability to tolerate a known pathology, disconnected from real thoughts and events, which tests the couple’s resilience.

Sexsomnia patients feel ashamed of their acts (unintentional and amnestic) and fear being exposed within relationships, whether familiar, social, or conjugal. On the other hand, their bed partners also suffer in feeling afraid of not sexually satisfying their partners, the fear of betrayal, and the shame of exposing themselves. The search for medical assistance, professionals who can help manage the parasomnia, may be compromised, and delayed under these circumstances.

Further, when considering the sexsomnia patient, a married woman who calls out for another man during nocturnal masturbation while sleeping with her husband, with children in the household, this presents a very unpleasant and problematic scenario, with potential harsh consequences that negatively affects all those involved.

Calling out a man’s name (viz. not her husband’s name) during sexsomnia behavior, was first reported by Dubessy et al.^6^ in a series of 17 patients in 2016. One of these cases, a 76-year-old woman, married for 50 years with the same person, experienced amnestic nocturnal masturbation for 3 years. This behavior and the verbalization caused psychological consequences such as shame and even suicidal ideation, as well as significant self-imposed changes to her social and familial routine.

In our case report, for the second time in literature, the patient called out for another man, and suffered significant psychosocial consequences, even after the sexsomnia diagnosis.

Shame and fear may be the primary reason that sexsomnia patients avoid treatment or even discuss the uncomfortable subject. In the Dubessy et al.^6^ case, the patient was reluctant to undergo couple’s therapy and, in the case reported here, the patient waited 10 years to seek medical assistance.

Suffering may be hidden not only by the patients themselves, but also by their bed partners and family members. To evaluate the extent to which this suffering could be ameliorated, deep understanding of the family dynamics is necessary.

Finally, there are occasional cases of sexsomnia that pose major diagnostic and therapeutic challenges^30^, underscoring the need for a multi-disciplinary approach to the patient.
